# Composition and contents of fatty acids and amino acids in the mycelia of *Lentinula edodes*

**DOI:** 10.1002/fsn3.3392

**Published:** 2023-06-01

**Authors:** Chang‐Xia Yu, Ya‐Ru Zhang, Yun‐Fei Ren, Yan Zhao, Xiao‐Xia Song, Huan‐Ling Yang, Ming‐Jie Chen

**Affiliations:** ^1^ Institute of Edible Fungi, Shanghai Academy of Agricultural Sciences, National Engineering Research Center of Edible Fungi, Key Laboratory of Edible Fungi Resources and Utilization (South) Ministry of Agriculture Shanghai China

**Keywords:** amino acid composition, fatty acid, *Lentinula edodes*, nutritional evaluation, proteins

## Abstract

With the global shortages of animal protein foods, mycoprotein as a low‐cost alternative source of protein by its high‐protein and low‐fat content has become a development trend. *Lentinula edodes* (*L. edodes*) is a healthy food with high protein and low fiber. This work evaluated the nutritional value of *L. edodes* mycelia, and determined the composition and contents of fatty acids and amino acids. Eleven saturated fatty acids (SFAs) and 12 unsaturated fatty acids (UFAs) were detected in the mycelia of *L. edodes*. The UFA content accounted for 75.7% and 73.1% of the total fatty acid content in the mycelia of strains 18 and 18N44, respectively. Linoleic acid was the major polyunsaturated fatty acid (PUFA) in the mycelia, accounting for 91.0% and 86.3% of the UFAs, respectively. The mycelia of the two strains contained 17 types of amino acids, and the essential amino acids were sufficient (357.92 ± 0.42 and 398.38 ± 4.52 mg/g pro, respectively), both close to the WHO/FAO reference protein pattern value. The most abundant essential amino acid was Lys, and the limiting amino acids were Met + Cys and Ile, respectively. The SRC values in the mycelia of the two strains were 68.07 and 54.86, and the EAAI values were 67.70 and 74.42, respectively, both being close to those of ovalbumin. It is concluded that *L. edodes* mycelia are rich in easily absorbed high‐quality proteins and PUFAs, and can be used as a source for meat analog required by vegetarians. This study provides a scientific basis for the further utilization of mycelial resources.

## Graphical abstract

Unsaturated fatty acids (UFAs) were dominant in the mycelia of *Lentinula edodes* strains 18 and 18N44, accounting for 75.7% and 73.1% of the total fatty acid content, respectively. Linoleic acid was the major PUFA in the mycelia, accounting for 91.0% and 86.3% of the UFAs in 18 and 18N44, respectively. The total amino acid and essential amino acid contents in the mycelia of strain 18 were 21.62% and 7.13%, respectively, which were significantly higher than those of the fruiting bodies of conventional strains of *L. edodes.* Lysine was the most abundant essential amino acid in the mycelia of *Lentinula edodes*, which could be used with cereal food to achieve balanced intake of amino acids.

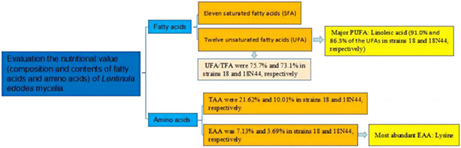

## 1 INTRODUCTION

“Artificial meat” made more attention by the shortages of animal protein food; it is mainly based on plant‐based meat products, and its main raw material (soy protein) itself has a strong fishy smell, and many of them contain lots of saturated fat, salt, and sugar. Therefore, it is necessary to discover more ways to get high‐quality, healthier protein. Edible mushrooms provide high‐quality proteins and present more productivity and biological efficiency than animals as a protein resource (Fabiane et al., 2018). These mushrooms are rich in dietary fiber, minerals, and vitamins and have a low lipid content, abundant amino acids, and a high proportion of polyunsaturated fatty acids (Jonathan et al., 2013). Owing to their high‐protein and low‐fat contents, mushrooms are excellent foods for low‐calorie diets (Bárbara et al., 2009). Therefore, it has better environmental and economic benefits than plant and animal proteins, and can be used as a low‐cost alternative source of protein (Gonzalez et al., 2020).

*Lentinula edodes* (*L. edodes*) Sing belongs to the fungal taxonomic groups, *Basidiomycetes*, *Agariciidae*, and *Pleurotus*. *Lentinula edodes* is not only rich in protein, minerals, and other nutrients but also has a variety of medicinal and health functions, such as antioxidant and antiviral activities (Wang et al., 2018). Mycoprotein has received extensive attention as a new type of protein material, with the deepening of the concept of healthy green diet and the expansion of the meat substitute market. *Lentinula edodes* has high nutritional value with high protein content in fruiting bodies. However, the long growth period of *L. edodes* and the difficulty in controlling the cultivation process and environmental conditions limit its wide application. Proteins can be obtained not only from the fruiting bodies of edible fungi but also from the mycelia. Proteins obtained from the mycelia by liquid fermentation technology could not only realize the standardized and annual production of products, shortens the production cycle, and improves safety and stability but also improve the yield of the products. Therefore, we explored the nutritional value of *L. edodes* mycelia to satisfy market demand.

Lipids are important energy substances, sources of essential fatty acids, and important components of living organisms, while fatty acids are the key components of lipids. The fatty acids, which are classified as saturated and unsaturated fatty acids, contained in mushrooms cannot be ignored. The importance of unsaturated fatty acid consumption, particularly that of long‐chain polyunsaturated fatty acids, for reducing blood cholesterol, as well as regulating cell physiology, has been widely demonstrated (Hossain et al., 2003). Although unsaturated fatty acids, such as linoleic and α‐linolenic acids, are essential for human metabolism, they are not produced by the human body (Mokochinski et al., 2015; Milgen & Dourmad, 2015). In addition, Yao et al. (2014) pointed out that polyunsaturated fatty acids in the diet have a unique regulatory effect on the mutagenesis of a variety of cancer cells. The research on fatty acids in mycelia of *L. edodes*, especially the types and contents of unsaturated fatty acids, is important for the exploitation and utilization of resources for mycelia.

Among edible fungi, *L. edodes* is known for its nutritional components and the pharmacological value of active compounds, especially the high protein and essential amino acid (EAA) levels (Bisen et al., 2010; Manzi et al., 1999). Because of its vital role in protein deposition, interaction with other amino acids, and other critical physiological roles (Ball et al., 2007), lysine is frequently used as a reference amino acid to estimate the requirements for other amino acids in a cultured species based on the ideal protein concept, when both essential amino acids and nonessential amino acids are limited (Vangaveti et al., 2015). This study used gas chromatography–mass spectrometry (GC–MS) and atomic absorption spectroscopy (AAS) to analyze and determine the types and contents of fatty acids and amino acids in the mycelia of two strains of *L. edodes*.

## 2 MATERIALS AND METHODS

### 2.1 Sample collection

Strain 18 of *L. edodes* is the main cultivated strain in Pingquan, Hebei Province. Strain 18N44 of *L. edodes* is a thermotolerant strain obtained by mutagenesis of strain 18, and compared with 18, it has stronger resistance to high temperature in the mycelium and seed stages (Wang et al., 2014).

### 2.2 Materials and instruments

PDA (potato dextrose agar) and PDB (potato dextros) media were purchased from BD Company, and concentrations of 39 and 24 g/L were used to prepare solid and liquid media, respectively.

All the reagents used in this study were of analytical grade. Hydrochloric acid and sulfuric acid were purchased from Sinopharm Chemical Reagent Co., Ltd. Methyl nonadecanoate (internal standard) was supplied by Sigma. The amino acid content was determined using an L‐8900 automatic amino acid analyzer (Hitachi Ltd.). The fatty acid content was determined by a 7890A‐5975C gas chromatography–mass spectrometer (Agilent Technologies).

### 2.3 Mycelia collection

The mycelia were subcultured on a PDA plate. After incubation for 10 days at 25°C, the culture was transferred to 250‐mL flasks containing 120 mL of PDB medium, and the flasks were incubated at 25°C with shaking at 150 rpm for 14 days. The mycelia were collected and dried at 60°C.

### 2.4 Preparation of samples used for fatty acid profiling

Mycelia (0.2 g) were added to a screw‐cap ampere tube with 1.0 mL of 5% H_2_SO_4_ and 5 μL of internal standard for fatty acid extraction. Nitrogen was blown into the tube every 10 s to remove air. The samples were refrigerated at 4°C for 10 min immediately after heating at 80°C for 90 min, and then transferred to a glass bottle. Pure water (0.5 mL) and hexane (1.0 mL) were added. Then, the tube was shaken for 20 s and centrifuged at 350× *g* for 10 min. After static layering, the upper layer was placed into an injection bottle, and GC–MS analysis was performed on the sample in the injection bottle (Mokochinski et al., 2015; Yu et al., 2019).

The chromatography parameters were as follows: chromatographic column: DB‐5 ms (30 m × 0.25 mm × 0.25 μm); injection volume: 1.00 μL; injection temperature: 270°C; split ratio: 1:5; carrier gas: helium (99.999%); flow rate: 1 mL/min; column temperature: 70°C for 5 min, 25°C/min to 200°C, 2°C/min to 240°C, 20°C/min to 300°C (keep consistent), and hold for 7 min; interface temperature: 280°C; ion source temperature: 230°C (keep consistent); quadrupole temperature: 150°C; ionization mode: EI, 70 eV; detector voltage: 2106 V; scan mode: full scan mode; and mass range (m/z): 33–500. The types of fatty acids were analyzed for alignments against the NIST2014 spectral library.

### 2.5 Amino acid extraction for automatic analysis of amino acids with AAS

The samples were freeze‐dried for 24 h and then ground to obtain powdered samples, from which hydrolyzed amino acids were extracted. Approximately 20 mg of each sample was suspended in 2 mL of 6 mol/L hydrochloric acid (HCl) and 2 μL of phenol. The ampere tubes were filled with nitrogen for 5 min, sealed, and hydrolyzed in a drying oven for 22–24 h. The hydrolysates were cooled and transferred to a 25‐mL volumetric flask. One milliliter of the supernatant was centrifuged at 12,000× *g* for 10 min and dried by a nitrogen blower. Then, 1 mL (0.02 mol) of HCl was added to the dried samples, and the samples were mixed in a shaker. The samples were filtered with a 0.45 μm cellulose filter membrane prior to analysis (Lee et al., 2011). Seventeen free amino acids were determined using an automatic amino acid analyzer (Yu et al., 2019).

### 2.6 Statistical analysis

Nutritional evaluation methods were used for amino acid analysis, including for determination of the essential amino acids (EAAs), amino acid score (AAS), chemical score (CS), ratio coefficient (RC), score of the RC (SRC), and essential amino acid index (EAAI). The AAS was calculated according to the method proposed by the FAO/WHO Expert Consultation (1989). The CS was calculated by the methods recommended by Seligson and Mackey (1984). The RC and SRC were calculated according to the method proposed by Zhu and Wu (1988). The EAAI was evaluated according to Oser (1951, 1959).

Analysis of variance (ANOVA) was performed with SPSS 19.0, and multiple comparisons were performed with Duncan's test.

## 3 RESULTS AND DISCUSSION

### 3.1 GC–MS analysis of the fatty acids in the mycelia of strains 18 and 18N44

**FIGURE 1:**
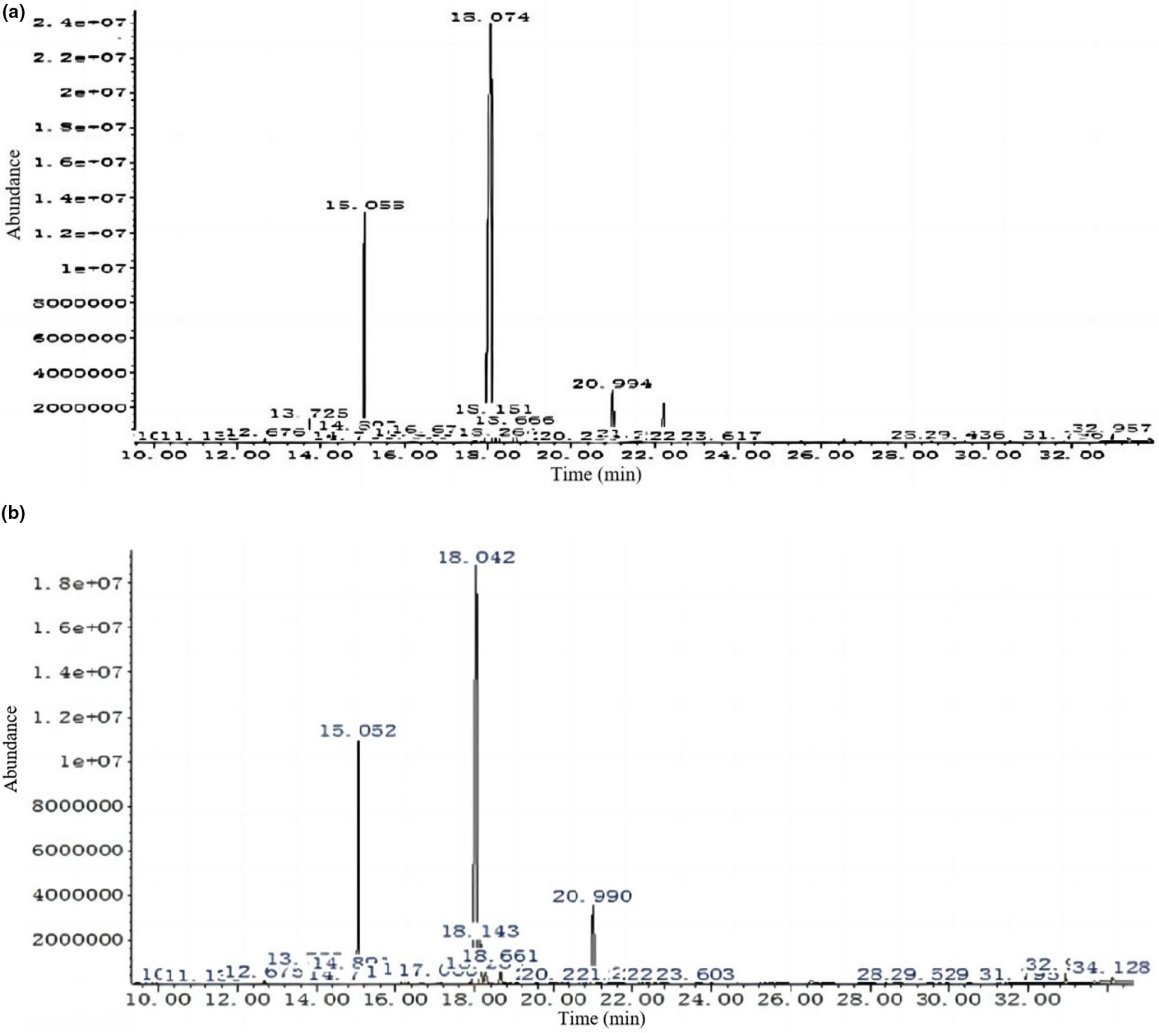
GC–MS analysis of the fatty acids in the mycelia of strains 18 and 18N44.

**FIGURE 2:**
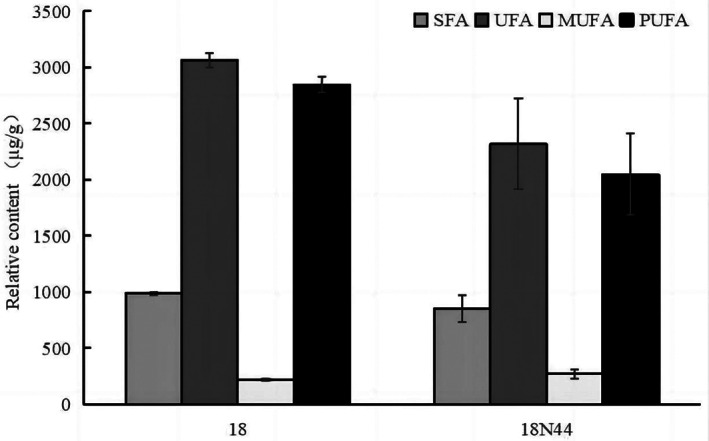
Fatty acid contents in the mycelia of strains 18 and 18N44.

The total ion chromatograms of the fatty acids in the mycelia of strains 18 and 18N44 are shown in Figure 1. GC–MS analysis revealed 11 saturated fatty acids (SFAs) and 12 unsaturated fatty acids (UFAs) in the mycelia of strains 18 and 18N44. However, the SFAs were not exactly identical. The unique SFAs were 2‐hydroxyoctadecanoic acid (2‐OH, C18:0) in the mycelia of strain 18, and 2‐hydroxyhexadecanoic acid (2‐OH, C16:0) and 2‐hydroxytetradecanoic acid in the mycelia of strain 18N44 (2‐OH, C24:0). Additionally, a chromatographic data processing system was used to calculate the fatty acid contents in the mycelia of strains 18 and 18N44, which are shown in Figure 2. The total fatty acid contents in the mycelia of strains 18 and 18N44 were 4049.0 and 3170.5 μg/g, respectively. The SFA and UFA contents in the mycelia of strain 18 were higher than those in the mycelia of strain 18N44, and the UFAs were dominant in the mycelia of both strains 18 and 18N44, which accounted for 75.7% and 73.1% of the total fatty acid content, respectively. The analysis of fatty acids analysis in the mycelia of *L. edodes* showed that the UFAs were at higher concentrations than SFAs. UFAs were dominated by polyunsaturated fatty acids (PUFAs), which accounted for 92.9% and 88.3% of the UFA content in the mycelia of strains 18 and 18N44, respectively. The ratio of UFA to SFA is also an important indicator for evaluating the nutritional value of edible fungi (Zhou et al., 2019), and the sample with higher USFA/SFA has higher health effects. The ratios of the parameter in the mycelia of strains 18 and 18N44 were 3.11 and 2.70, respectively, indicating that strain 18 had a higher nutritional value than strain 18N44.

### 3.2 Analysis of six relatively major fatty acids in the mycelia of *Lentinula edodes* strains 18 and 18N44

**FIGURE 3:**
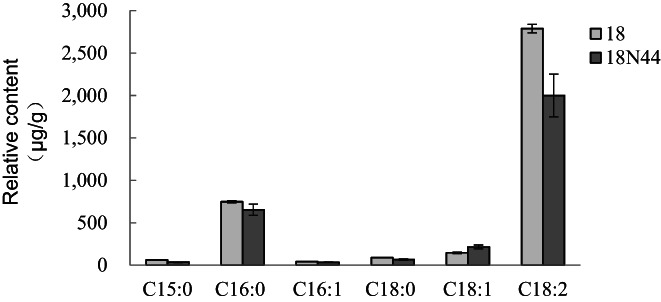
Relative contents of six major fatty acids in the mycelia of strains 18 and 18N44.

The relative contents of six fatty acids were high in the mycelia of strains 18 and 18N44, representing 95.6% and 94.6% of the total fatty acid contents of the two strains, respectively. The six major fatty acids identified were as follows: pentadecanoic acid (C15:0), palmitic acid (C16:0), palmitoleic acid (C16:1), octadecanoic acid (C18:0), oleic acid (C18:1), and linoleic acid (C18:2); the contents are shown in Figure 3, and linoleic acid content was the most abundant among all the components. Specifically, the relative content of oleic acid in the mycelia of strain 18N44 was higher than that in the mycelia of strain 18, while the relative contents of the other five fatty acids in strain 18N44 were all lower than those in strain 18. The correlation analysis of the six major fatty acid contents is shown in Table 1. There was a certain correlation among the contents of each fatty acid. Except for the content of oleic acid, which was negatively correlated with those of the other five fatty acids, the contents of the other fatty acids were all positively correlated, which was consistent with the above results.

**TABLE 1:** Correlation analysis of six major fatty acid components in the mycelia of *Lentinula edodes.*

	C15:0	C16:0	C16:1	C18:0	C18:1	C18:2
C15:0	1.000	0.785	0.855*	0.903*	−0.663	0.956**
C16:0	0.785	1.000	0.923**	0.801	−0.116	0.928**
C16:1	0.855*	0.923**	1.000	0.769	−0.331	0.923**
C18:0	0.903*	0.801	0.769	1.000	−0.371	0.904*
C18:1	−0.663	−0.116	−0.331	−0.371	1.000	−0.448
C18:2	0.956**	0.928**	0.923**	0.904*	−0.448	1.000

* indicates significant correlation at the level of 0.05.

** indicates significant correlation at the level of 0.01.

In the mycelia of strains 18 and 18N44, the major SFA was palmitic acid, with contents of 746.8 μg/g (18.4% of the total fatty acids; 75.8% of the SFAs) and 652.0 μg/g (20.6% of the total fatty acids; 76.4% of the SFAs), respectively. The major monounsaturated fatty acid (MUPF) was oleic acid; in strains 18 and 18N44, the oleic acid contents were 146.2 μg/g (3.6% of the total fatty acids; 4.8% of the UFAs) and 214.0 μg/g (6.7% of the total fatty acids; 9.2% of the UFAs), respectively (Figure 3). Hargrove et al. (2001) pointed out that dietary MUPFs are another consideration for low‐fat diets and can decrease blood cholesterol levels and regulate immune function. The major PUFA was linoleic acid, with contents of 2788.8 μg/g (68.9% of the total fatty acids; 91.0% of the UFAs) and 2000.2 μg/g (63.1% of the total fatty acids; 86.3% of the UFAs) in strains 18 and 18N44, respectively. The ratio of linoleic acid in total fatty acids was higher than that of seven kinds of edible mushrooms (13%–59%; Yilmaz et al., 2006) and slightly lower than that of *L. edodes* fruiting body. The low‐fat content of mushrooms is a nutritional advantage, and their lipid components, as important metabolites, have beneficial nutritional value (Marekov et al., 2012). Linoleic acid is an essential fatty acid in the human body, which has the functions of lowering serum cholesterol levels and preventing myocardial infarction, diabetes, and arteriosclerosis (Castro‐webb et al., 2012). The results showed that the mycelia of *L. edodes* were low in fat and high in unsaturated fatty acids, and could prevent the occurrence of cardiovascular diseases.

### 3.3 Analysis of the amino acid contents in strains 18 and 18N44

The amino acid profile and protein quality of the mycelia of strains 18 and 18N44 were systematically determined and evaluated. Seventeen amino acids detected in *L. edodes* strains 18 and 18N44 clearly differed between the two strains (The sample was hydrolyzed with hydrochloric acid, which could destroy tryptophan, so tryptophan was not detected in this test.). The total amino acid (TAA) and essential amino acid (EAA) contents in the mycelium of strain 18 were 21.62% and 7.13%, respectively, which were higher than those of *L. edodes* from different producing areas (14.39–18.71 g/100 g TAA and 4.68–6.33 g/100 g EAA, respectively; Li et al., 2020). The values were also higher than those of *L. edodes* strains 808 (14.99 g/100 g TAA and 5.11 g/100 g EAA) and ww808 (18.30 g/100 g TAA and 5.76 g/100 g EAA; Yu et al., 2019). The results showed that the contents of total amino acid and essential amino acid in the mycelia of strain 18 were rich, which were significantly higher than those of the fruiting bodies of conventional varieties of *L. edodes*. In addition, the essential amino acid contents of *Fusarium venenatum* which is used by the Quorn brand name (Miller & Dwyer, 2001) and *Fusarium strain flavolapis* used by the company Nature's Fynd (Furey et al., 2022) were 4.59 and 5.4 g/100 g, respectively; these data indicated that strain 18 also had a higher essential amino acids content than the above two commercial strains. The TAA and EAA contents of the mycelium of strain 18N44 were 10.01% and 3.69%. The two strains had similar amino acid compositions. Glutamic acid and arginine were among the most common amino acids, while cysteine was rarely detected (Table 2). Glutamic acid, a major neurotransmitter and the most abundant free amino acid in the brain, is synthesized in the brain because the passage of glutamate from the blood to the brain is effectively prevented by the blood–brain barrier (Fernstrom, 2018).

**TABLE 2:** Amino acid composition of the proteins in strains 18 and 18N44 (%).

	18 (%)	18N44 (%)
Asp	1.73 ± 0.05	0.74 ± 0.03
Thr	1.01 ± 0.03	0.50 ± 0.02
Ser	1.04 ± 0.03	0.47 ± 0.02
Glu	4.93 ± 0.15	1.54 ± 0.08
Gly	0.89 ± 0.03	0.42 ± 0.02
Ala	1.17 ± 0.04	0.60 ± 0.02
Cys	0.08 ± 0.00	0.05 ± 0.00
Val	1.05 ± 0.03	0.52 ± 0.03
Ile	0.62 ± 0.02	0.31 ± 0.01
Leu	1.29 ± 0.04	0.53 ± 0.02
Tyr	0.54 ± 0.02	0.21 ± 0.02
Phe	0.83 ± 0.03	0.37 ± 0.02
Lys	1.91 ± 0.07	1.21 ± 0.04
His	0.84 ± 0.07	0.39 ± 002
Arg	2.45 ± 0.08	1.53 ± 0.05
Met	0.42 ± 0.01	0.25 ± 0.01
Pro	0.82 ± 0.03	0.37 ± 0.02
TAAs	21.62 ± 0.17	10.01 ± 0.14
EAAs	7.13 ± 0.05	3.69 ± 0.06
NEAAs	14.49 ± 0.04	6.32 ± 0.04
E/T	32.98 ± 0.00	37.20 ± 0.01
E/N	49.21 ± 0.00	59.29 ± 0.03

*Note*: Values were calculated from triplicate samples.

Abbreviations: EAAs, essential amino acids; E/N, essential/nonessential amino acids; E/T, essential/total amino acids; NEAAs, nonessential amino acids; TAAs, total amino acids.

The E/T values of the two strains were 32.98% and 37.20%, respectively, which were close to the ideal protein model value (>40%) proposed by the FAO/WHO, and the E/N values were 49.21% and 59.29%, respectively, which were also close to the ideal protein standard (>60%), indicating that the proteins in the mycelia of the two strains were an excellent source of protein nutrition. Therefore, they could be used together with other foods to improve the quality of amino acids consumed.

### 3.4 Analysis of the contents of taste‐active amino acids

The main bitter amino acids are valine, leucine, isoleucine, methionine, arginine, and arginine (Gurkirat et al., 2012). The taste‐active amino acid contents and proportions relative to total amino acids (TAAs) in the mushroom strains are shown in Table 3. The relative contents of the taste‐active amino acids in the mycelia of strain 18 were in the order bitter amino acids ≥ umami amino acids > sweet amino acids > aromatic amino acids, while in the mycelia of strain 18N44, they were in the order bitter amino acids > sweet amino acids > umami amino acids > aromatic amino acids. The contents of sweet amino acids and bitter amino acids in strain 18N44 were higher than those in strain 18, but the contents of umami amino acids were nearly 10.0% lower than those in strain 18.

**TABLE 3:** Taste‐active amino acid contents and proportions relative to total amino acids (TAAs) in strains 18 and 18N44 (%).

Amino acid		18	18N44
Sweet amino acids	Content	4.93 ± 0.16	2.36 ± 0.19
	Proportion	22.81	23.74
Umami amino acids	Content	6.66 ± 0.21	2.28 ± 0.13
	Proportion	30.83	22.12
Aromatic amino acids	Content	1.37 ± 0.05	0.58 ± 0.02
	Proportion	6.33	5.78
Bitter amino acids	Content	6.66 ± 0.24	3.52 ± 0.09
	Proportion	30.84	35.63

### 3.5 Nutritional evaluation of the amino acids in *Lentinula edodes*

#### 3.5.1 Composition of essential amino acids in *Lentinula edodes*

The nutritional value of proteins mainly depends on the types and quantity and composition ratio of essential amino acids. The two strains exhibited abundant essential amino acids, with total contents of 357.92 ± 0.42 and 398.38 ± 4.52 mg/g pro in strains 18 and 18N44, respectively (Table 4). The total content of essential amino acids in strain 18N44 was higher than that in strain 18, and both strain 18 and strain 18N44 showed superior essential amino acid contents to the WHO/FAO reference protein pattern value (350 mg/g pro). However, the contents of essential amino acids in the two strains were inferior to the ovalbumin model value (497 mg/g pro).

**TABLE 4:** Essential amino acid composition of proteins in strains 18 and 18N44 (mg/g pro).

Amino acid	18	18N44	WHO/FAO	Ovalbumin
Thr	46.53 ± 0.13	50.32 ± 0.44	40	51
Lys	88.20 ± 0.43	122.04 ± 2.93	55	64
Leu	59.60 ± 0.09	52.97 ± 0.23	70	88
Ile	28.88 ± 0.11	31.59 ± 0.83	40	66
Met + Cys	22.84 ± 0.61	31.88 ± 0.69	35	55
Phe+Tyr	63.27 ± 0.26	57.77 ± 0.81	60	100
Val	48.60 ± 0.04	51.81 ± 1.16	50	73
Total	357.92 ± 0.42	398.38 ± 4.52	350	497

In this study, a relatively high content of lysine was observed among the essential amino acids of *L. edodes* 18 and 18N44 mycelial proteins, which was superior to that of the ovalbumin model. Lysine promotes human development, enhances immunity, and improves the function of central nervous tissue. Lysine is the first‐limiting amino acid in cereals and the first essential amino acid in the human body, with important functions in enhancing immunity and promoting human growth and development and benefits for central nervous system tissue (Shimomura et al., 2014). Lysine is low in abundance in wheat and rice and is easily destroyed during processing. Lysine was also considered one of the limiting amino acids in plant‐derived proteins that could be used for supplementation to vegetarians. Therefore, *L. edodes* mycelial proteins can be used with cereal food or other plant‐derived food to achieve balanced intake of amino acids and compensate for the lack of lysine when cereals are the main food.

#### 3.5.2 Evaluation of the nutritional profile of essential amino acids in the mycelia of *Lentinula edodes*

The AAS, which represents the percentage of a single essential amino acid in a tested protein sample in relation to the corresponding amino acid in the WHO/FAO reference protein pattern, is a major index to evaluate the nutritional value of the amino acid compositions. According to the AAS values, Met + Cys was the first‐limiting amino acid in strain 18, while Leu was the first‐limiting amino acid in strain 18N44 (Table 5). The composition and proportion of essential amino acids in strain 18N44 were closer to the amino acid composition required by humans than those in strain 18. It is worth noting that the AASs of Lys in the two strains were higher than the model value. Lysine exhibits important functions such as those in promoting growth and development. Food crops are enriched with sulfur‐containing amino acids but lack Lys and Leu; thus, these foods can be used in combination with *L. edodes* strain 18.

**TABLE 5:** Nutritional profile of amino acids in the proteins of strains 18 and 18N44.

	AAS (%)		CS (%)		RC	
	18	18N44	18	18N44	18	18N44
Thr	116.32 ± 0.32	125.81 ± 1.64	91.23 ± 0.25	98.68 ± 1.28	1.16 ± 0.00	1.11 ± 0.03
Lys	160.37 ± 0.79	221.89 ± 6.92	137.82 ± 0.68	190.68 ± 0.59	1.60 ± 0.01	1.96 ± 0.02
Leu	85.14 ± 0.14	75.67 ± 0.43	67.72 ± 0.11	60.19 ± 0.34	0.85 ± 0.00	0.67 ± 0.03
Ile	72.20 ± 0.26	78.98 ± 0.28	43.76 ± 0.15	47.86 ± 0.17	0.72 ± 0.00	0.70 ± 0.01
Met + Cys	65.27 ± 1.74	91.09 ± 0.23	41.53 ± 0.11	57.97 ± 0.14	0.65 ± 0.01	0.80 ± 0.06
Phe + Tyr	105.44 ± 0.43	96.29 ± 1.81	63.27 ± 0.26	57.77 ± 1.08	1.05 ± 0.00	0.85 ± 0.05
Val	97.20 ± 0.08	103.61 ± 0.31	66.58 ± 0.05	70.97 ± 0.21	0.97 ± 0.00	0.92 ± 0.01

The amino acid composition of egg protein is the most similar to that of proteins recommended for human consumption, and egg protein is often used as a reference in experiments and research (Kayode et al., 2015). The CS was determined with the method recommended by the FAO/WHO Expert Consultation (1989) for determining the content of an essential amino acid in a tested sample relative to the content of the corresponding essential amino acid in the standard ovalbumin reference protein. According to the CS values, Met + Cys was the first‐limiting amino acid in strain 18, while Ile was the first‐limiting amino acid in strain 18N44 (Table 5). Compared with the egg protein model, the balance and content of essential amino acids in the mycelia of *L. edodes* protein were slightly inferior to those in egg protein. Although the nutritional value of *L. edodes* mycelial proteins was not as good as that of egg protein, they were still a high‐quality protein resource and could be used as an auxiliary ingredient for nutritional matching with other foods.

The RC values of amino acids in the mycelia of strains 18 and 18N44 were 0.65–1.60 and 0.67–1.96, respectively. Lys and Met + Cys and Lys and Leu deviated positively and negatively from the amino acid equilibrium spectra of the two strains, respectively, and Val was closest to 1.00. The SRC values for the mycelia of strains 18 and 18N44 were 68.07 and 54.86, respectively. The values were higher than those of some grain crops, such as sorghum (47.33) and millet (53.15), close to those of rice (70.50) and wheat (72.47), and slightly inferior to those of eggs (81.22), indicating that the protein nutritional value of *L. edodes* mycelia is balanced and has broad prospects for development and utilization.

#### 3.5.3 EAAI values in the mycelia of *Lentinula edodes*

The EAAI values of the proteins in strains 18 and 18N44 were 67.70 ± 0.33 and 74.42 ± 0.76, respectively. The data obtained from this research revealed that the proteins of *L. edodes* mycelia exhibited a relatively balanced amino acid content, showing that they could serve as a favorable source of proteins with suitable proportions.

This study evaluated the amino acid composition and nutritional evaluation of the mycelia of different strains of *L. edodes* (18 and 18N44) and found that the nutritional composition of the different strains was not the same, potentially because 18N44 is a mutant derived from strain 18. Although strain 18N44 was resistant to high temperatures in the mycelium and fruiting stages (Wang et al., 2014), the structures of proteins may change during mutagenesis, altering the amino acid composition and influencing the amino acid quality. In this research, the amino acid contents of strain 18 were higher than those of strain 18N44, but the proportion of essential amino acids was lower. The nutritional value of proteins in strain 18N44 was more abundant. The mycelial amino acid scores of the two strains were higher than that of *Coprinus comatus* (59.6), *Agaricus blazei* Murrill (33.5), and *Auricularia auricula* (54.5; Jiang et al., 2009), which have high‐protein values. It is concluded that the mycelia of *L. edodes* are rich in high‐quality proteins that are easily absorbed and that are recommended to be combined with cereals in the diet for balanced consumption of amino acids.

## 4 CONCLUSION

This research studied the amino acids and fatty acids in the mycelia of *L. edodes* strains 18 and 18N44 to explore their nutritional value and to provide a reference for selecting foods to address nutrient deficiencies in specific populations (Song et al., 2019). The study provides a scientific basis for further utilization of mycelia resources. It is expected that mycelium of *L. edodes* had a potential advantage to be developed as an available protein source in addition to the various health benefits, and as a safe and good‐taste edible mushroom, *L. edodes* could be developed as the basic material for lysine supplement to the specific population whose dietary predilection is mainly plant species. It is also expected by lower fat and higher amino acids, making it potentially suitable for lower calorie “artificial meat,” possessing excellent flavor quality when compared to those made with soy proteins.

## AUTHOR CONTRIBUTIONS

**Changxia Yu:** Formal analysis (equal); methodology (equal); writing – original draft (equal). **Yaru Zhang:** Formal analysis (equal); writing – original draft (equal). **Yunfei Ren:** Data curation (equal); methodology (equal). **Yan Zhao:** Project administration (equal); supervision (equal); writing – review and editing (equal). **Xiaoxia Song:** Data curation (equal); resources (equal). **Huanling Yang:** Methodology (equal); resources (equal). **Mingjie Chen:** Funding acquisition (equal); project administration (equal); supervision (equal).

## CONFLICT OF INTEREST STATEMENT

The authors declare that they have no conflicts of interest.

## Data Availability

The data that support the findings of this study are available from the corresponding author on request.
